# Aptamer-functionalized graphene quantum dots combined with artificial intelligence detect bacteria for urinary tract infections

**DOI:** 10.3389/fcimb.2025.1555617

**Published:** 2025-04-16

**Authors:** Kun Li, Shiqiang Fang, Tangwei Wu, Chao Zheng, Yi Zeng, Jinrong He, Yingmiao Zhang, Zhongxin Lu

**Affiliations:** ^1^ Department of Medical Laboratory, The Central Hospital of Wuhan, Tongji Medical College, Huazhong University of Science and Technology, Wuhan, China; ^2^ Cancer Research Institute of Wuhan, The Central Hospital of Wuhan, Tongji Medical College, Huazhong University of Science and Technology, Wuhan, China; ^3^ Hubei Provincial Engineering Research Center of Intestinal Microecological Diagnostics, Therapeutics, and Clinical Translation, Wuhan, China

**Keywords:** graphene quantum dots, artificial intelligence, urinary tract infections, biosensor, *Escherichia coli*

## Abstract

**Objectives:**

Urinary tract infection is one of the most prevalent forms of bacterial infection, and prompt and efficient identification of pathogenic bacteria plays a pivotal role in the management of urinary tract infections. In this study, we propose a novel approach utilizing aptamer-functionalized graphene quantum dots integrated with an artificial intelligence detection system (AG-AI detection system) for rapid and highly sensitive detection of *Escherichia coli* (*E. coli*).

**Methods:**

Firstly, graphene quantum dots were modified with the aptamer that can specifically recognize and bind to *E. coli*. Therefore, the fluorescence intensity of graphene quantum dots was positively correlated with the concentration of *E. coli*. Finally, the fluorescence images were processed by artificial intelligence system to obtain the result of bacterial concentration.

**Results:**

The AG-AI detection system, with wide linearity (10^3^-10^9^ CFU/mL) and a low detection limit (3.38×10^2^ CFU/mL), can effectively differentiate between *E. coli* and other urinary tract infection bacteria. And the result of detection system is in good agreement with MALDI-TOF MS.

**Conclusions:**

The detection system is an accurate and effective way to detect bacteria in urinary tract infections.

## 1 Introduction

Urinary tract infection (UTI) is one of the common routes of bacterial infection in humans (Masajtis-Zagajewska and Nowicki, 2017). Studies have shown that more than 50% of women will experience at least one UTI in their lifetime (Jung et al., 2022). Thus, it is more pronounced in the female population (Dielubanza and Schaeffer, 2011). Most urinary tract infections are caused by bacteria in the urinary tract, and the organisms enter the urethra through the urine and subsequently enter the bladder cavity. Once inside the bladder, bacteria adhere to the bladder epithelial cells via adhesin-mediated mechanisms, rapidly proliferate, and cause bladder infection and inflammatory responses. When the infection is confined to the bladder, it is called cystitis. When the infection spreads to the kidneys, it is called pyelonephritis. If not treated properly, the bacteria can spread from the kidneys into the bloodstream, which may further lead to sepsis under systemic inflammation (Rahman Fink et al., 2018; Cao et al., 2023; Liu et al., 2023; Wang et al., 2023). Therefore, simple and accurate methods are urgently needed for identification different uropathogenic organisms. These novel measurements will be beneficial to accurate diagnosis and treatment of urinary tract infections.

The initial diagnosis of urinary tract infection is based on clinical symptoms including dysuria, dyspareunia, and urgency. Urine test strips are used to detect nitrite and leukocyte esterase, indicating bacteriuria and pyuria, respectively (Masajtis-Zagajewska and Nowicki, 2017; Coulthard, 2019). However, in the presence of non-nitrite-producing pathogens, urine test strips may produce false-negative results. The recognized gold standard for the diagnosis of bacterial urinary tract infections is *in vitro* urine culture (Capstick et al., 2024). However, identification of bacteria requires experienced examiners and the results varies from examiner to examiner. Compared to current urine culture methods, Molecular and proteomic techniques have improved detection efficiency and clinical outcomes, such as mass spectrometry, fluorescence *in situ* hybridization (FISH), and polymerase chain reaction (PCR) (Kumar et al., 2016). However, these technologies are expensive and difficult to popularize. Thus, there is still an urgent need to develop a new method to identify bacteria in urine of UTI patients.

Aptamer is a single-stranded DNA or RNA molecule that has an indeterminate conformation in solution (Kinghorn et al., 2017; Röthlisberger and Hollenstein, 2018). When a target molecule is present, the aptamer undergoes adaptive folding and binds to the target molecule with high affinity through hydrogen bonding, hydrophobic forces, and van der Waals forces. Aptamers are similar to antibodies, but they have a simpler chemical structure than antibodies and are more easily modified. In addition, aptamer is cheaper than antibody (Arao et al., 1999; Nanda and Juthani-Mehta, 2009; Horváth et al., 2020). Therefore, it is widely used in the detection of bacteria, proteins, exosomes, and neurotransmitters.

Quantum dots are low-dimensional semiconductor materials with a diameter of typically 2-20 nm, characterized by a broad excitation spectrum, high photochemical stability, and long fluorescence lifetime (Wang et al., 2020; Sun et al., 2023). Carbon quantum dots (CQDs) are fluorescent carbon nanomaterials (Rani et al., 2020; Walther et al., 2020), generally with a diameter of less than 10 nm, while graphene quantum dots (GQDs) are a subset of carbon quantum dots derived from graphene or graphene oxide (Lu et al., 2019). Compared with other quantum dots such as germanium quantum dots, cadmium sulfide quantum dots, and cadmium selenide quantum dots, GQDs exhibit tunable fluorescence properties, excellent photostability, good biocompatibility, low toxicity, and electrical conductivity. Therefore, they are widely used in the fields of drug transportation, bioimaging, and biosensing (Lee et al., 2020; Zhao et al., 2020; Chen et al., 2021; Huang et al., 2021).

In recent years, artificial intelligence, as a digital technology, has been rapidly developed (Maheshwari et al., 2023; Newby et al., 2023; Schattenberg et al., 2023). The applications of the technology are becoming more mature in the field of data processing, image processing, and so on (Brameier et al., 2023; Alsulimani et al., 2024; Jacot et al., 2024; Shickel and Bihorac, 2024). It is indicated that artificial intelligence is accurate, efficient, and flexible. In addition, artificial intelligence has the advantage of fast computing speed. It will play a vital role in transformation of traditional industries when the technology is applied across other fields. Especially, in the field of image recognition, machine learning has got great progress. Therefore, our research group construct an image-based medical judgment method, which could effectively recognize the target bacteria in more complex environments. Meanwhile, the subjective errors of manual observation will be eliminated due to the quantified results generated by the objective computational algorithms.

**Figure 1:**
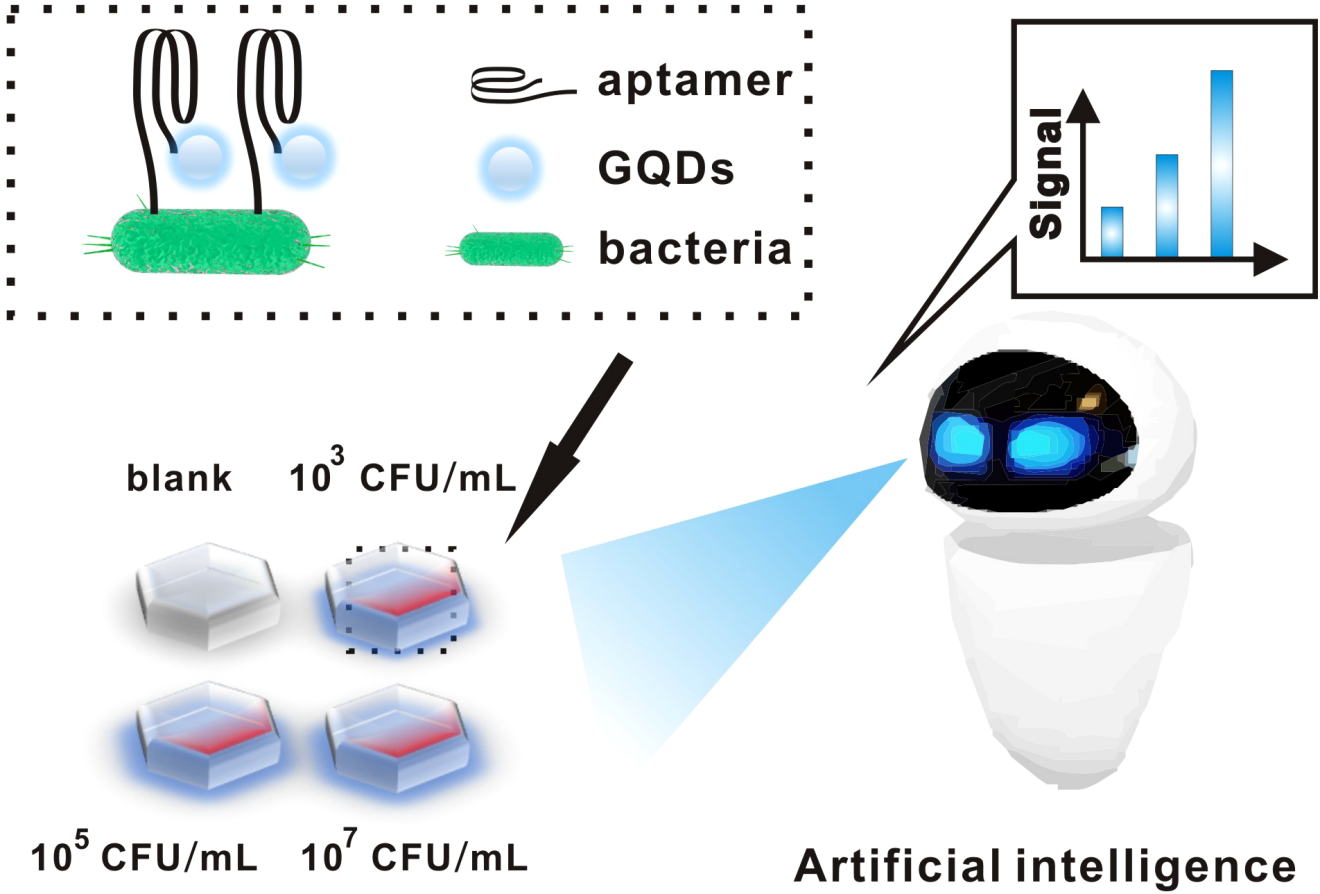
Schematic diagram of aptamer-functionalized GQDs combined with an artificial intelligence detection system for the detection of bacteria.

As shown in **Figure 1**, we fixed the bacteria on the slide and then added the aptamer-GQDs complex dropwise on the slide. Among them, aptamers can bind specifically to *E. coli*, and the fluorescence intensity of GQDs is positively correlated with the concentration of *E. coli*. At the same time, the artificial intelligence model begins to work. First of all, the fluorescence intensity of GQDs is converted into digital information by using image processing technology. Subsequently, the information-processed image features are analyzed by calculation module. Finally, accurate and objective result is outputted by detection platform for accurate diagnosis of urinary tract infections.

## 2 Materials and methods

### 2.1 Materials

Carboxylated GQDs were ordered from XFNANO (Nanjing, China). 1-Ethyl-3-(3-dimethyllaminopropyl) carbodiimide (EDC, 98%), and N-hydroxy-sulfosuccinimide (sulfo-NHS, 98.5%) were provided by Sigma-Aldrich (Shanghai, China). The DNA and DNA-Cy5 used in this study were provided by Sangon Biotech (DNA sequences: CCATGAGTGTTGTGAAATGTTGGGACACTAGGTGGCATAGAGCCG). The turbidimeter and turbidimetric tubes were provided by BD, USA. The type strain of *E. coli* is ATCC 25922.

### 2.2 Preparation of functionalized probes for GQDs

Similar to the previously reported method (Li et al., 2022; Li et al., 2023), we first configured 1 mg/mL of carboxylated graphene quantum dots, and then mixed 100 µL of GQDs with 100 µL of EDC/NHS (the concentration of EDC was 10 mg/mL and the concentration of NHS was 2.5 mg/mL), and incubated them for 30 min at room temperature away from light to achieve the activation of carboxyl groups. Then, we took 100 µL of the activated GQDs solution and mixed it with the aptamer at a concentration of 1 µM in equal proportions and incubated it for 2h to complete the preparation of GQDs functionalized probes.

### 2.3 *E. coli* immobilized on a slide

We added 5 µL of *E. coli* suspensions of different concentrations dropwise onto the slides, and after they dried naturally, the slides were fixed on a 75°C roaster for 10 min to complete the *E. coli*.

### 2.4 Functionalized GQDs Detect *E. coli*

The pre-made GQDs-aptamer complex (10 µL) was added dropwise to *E. coli* slides and incubated for 30 min at room temperature away from light to fully bind the aptamer to *E. coli*, followed by rinsing the slides with 1×PBS and deionized water to remove unbound GQDs-aptamer. Finally, the slides were placed under a fluorescence microscope for observation.

### 2.5 Artificial intelligence processing of inspection results

Images under a fluorescence microscope are judged using artificial intelligence. Firstly, the fluorescence image is converted into a grey scale image, followed by median filtering and binary in turn, and then the length and width of the image are read out. Finally, each pixel point in the picture is read, and finally the fluorescence intensity of graphene quantum dots in the picture, is converted into a numerical signal. Additionally, we conducted an ablation study on the AG-AI detection system, which demonstrated that the system possesses a high level of accuracy (**Table 1**). For more detailed information, please refer to the Supporting Information (SI).

**Table 1:** The ablation study of AG-AI detection system.

Experimental Conditions	Accuracy (%)
Complete Model	96.7
Remove Red Channel	93.3
Remove Green Channel	90
Remove Blue Channel	83

## 3 Results

### 3.1 Characterization of aptamer-functionalized GQDs

**Figure 2:**
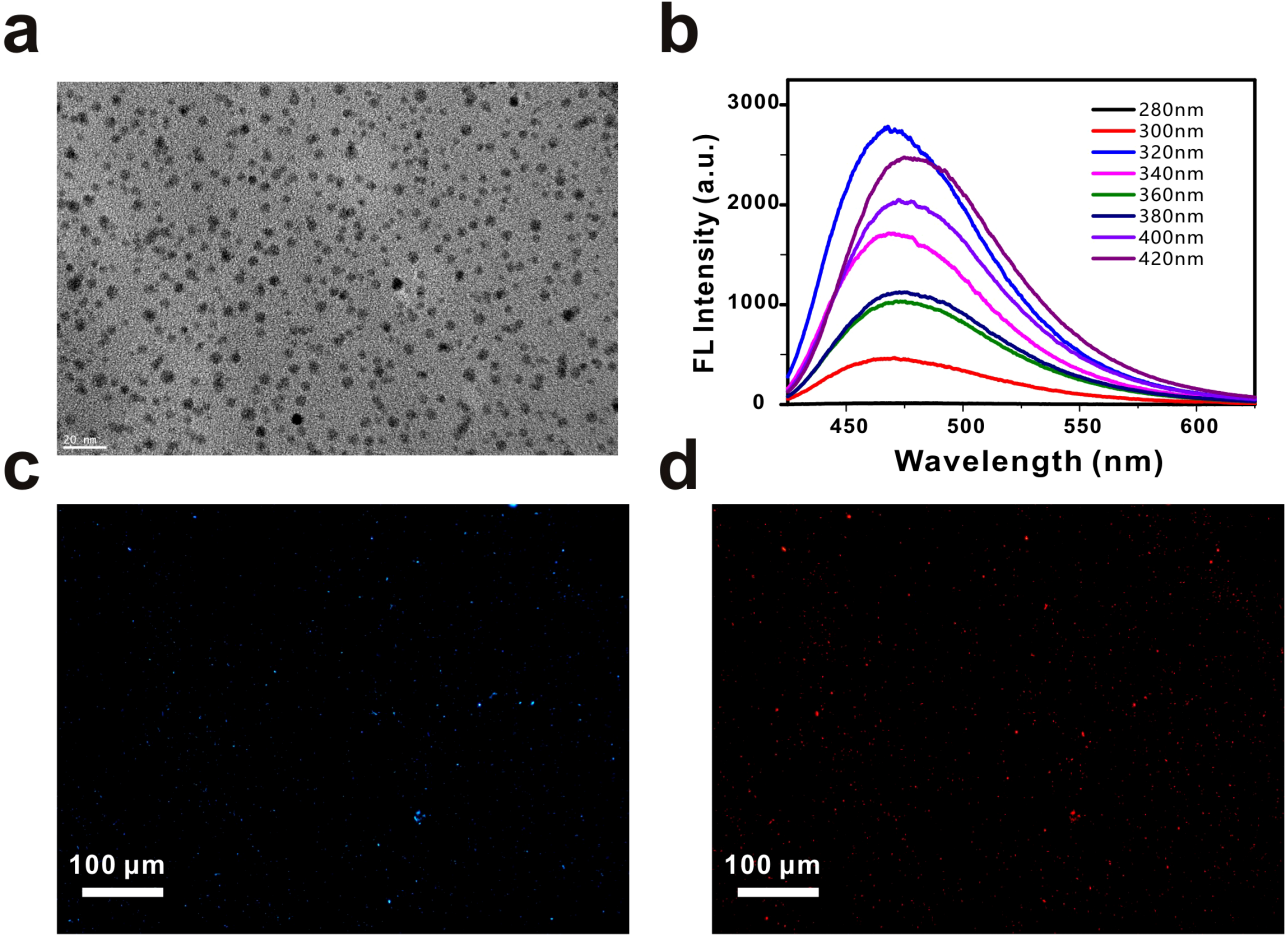
The characterization of GQDs. **(a)** TEM image of the GQDs. Scale bar, 20 nm. **(b)** Fluorescence spectra of GQDs from 280 nm to 420 nm. **(c)** Fluorescence images of Escherichia coli captured by aptamer-functionalized GQDs. Scale bar, 100 µm. **(d)** Fluorescence images of Escherichia coli captured by Cy5-modified GQDs. Scale bar, 100 µm.

Firstly, the size of the GQDs was measured by transmission electron microscopy. As shown in **Figure 2a**, the GQDs were uniformly dispersed and had an average particle size of about 4 nm (**Supplementary Figure S2**). **Figure 2b** shows the fluorescence emission spectra of the GQDs at excitation wavelengths of 280 nm-420 nm. At the excitation wavelengths of 280 nm-420 nm, the GQDs can emit blue light. When the excitation wavelength is at 320 nm, the fluorescence emission spectrum of GQDs reaches the maximum. This proves that GQDs can emit blue light (475 nm) under UV excitation. Next, we modified the other end of the aptamer with a Cy5 fluorescent group, and as in the previous method, the GQDs carrying the Cy5 fluorescent group were used to bind to *E. coli* immobilized on slides. **Figure 2c** is the picture under UV excitation light, where dense blue fluorescent dots can be seen, and **Figure 2d** is the picture in the same field of view under yellow-green light excitation, where red fluorescence of Cy5 appears in the same position. This proves that aptamer-functionalized GQDs are feasible to detect *E. coli*.

### 3.2 Aptamer-functionalized GQDs combined with artificial intelligence for detection of *E. coli*

**Figure 3:**
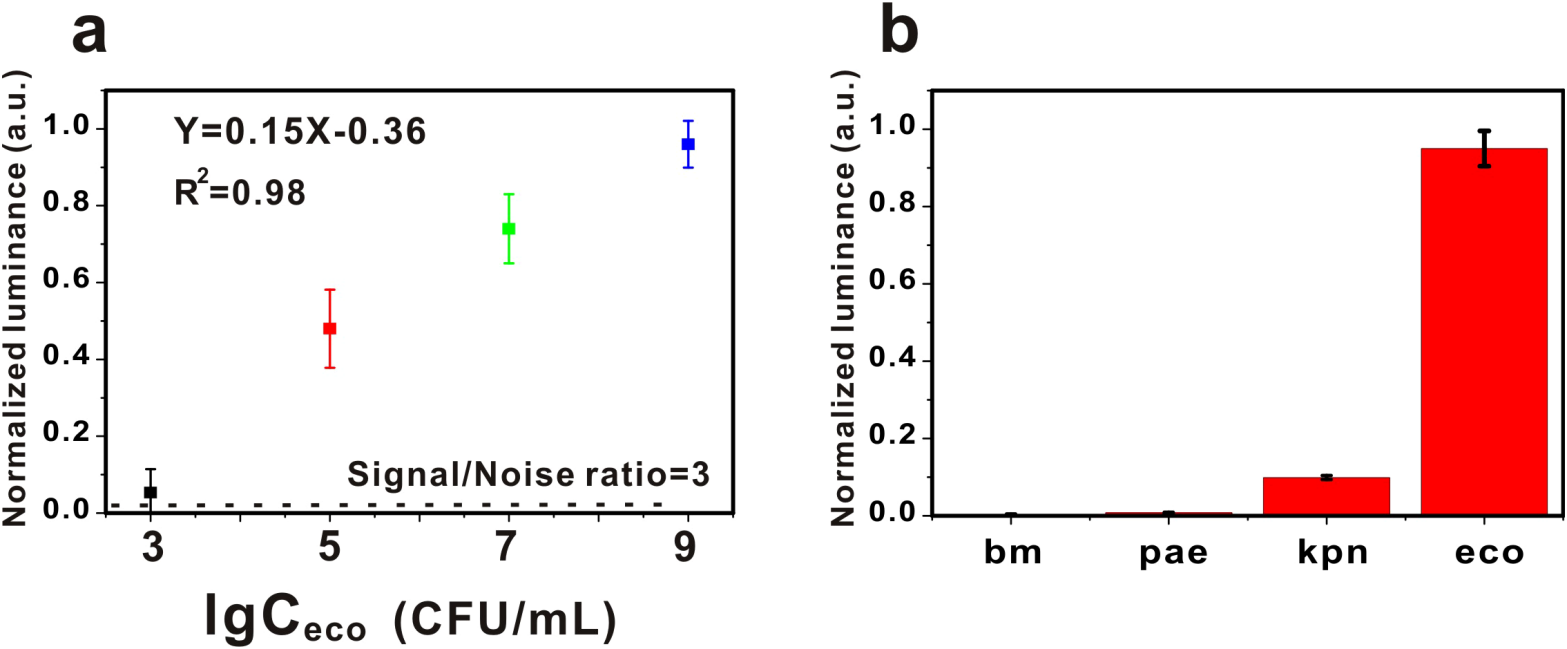
**(a)** The sensitivity of the AG-AI detection system for the detection of *Escherichia coli*. Error bars represent the standard errors (n = 3). The black dashed line represents the 3-fold noise level (∼0.02) from the blank. **(b)** The specificity of the AG-AI detection system for the detection of *Escherichia coli*. bm: *Acinetobacter baumannii;* pae: *Pseudomonas aeruginosa;* kpn: *Klebsiella pnenmoniae;* eco: *Escherichia coli.* Error bars represent the standard errors (n = 3).

A python algorithm was integrated into the AP-GQDs detection module and the sensitivity and specificity of this detection system was investigated. As shown in **Figure 3a**, we detected *E. coli* at different concentrations, obtained fluorescence images at different *E. coli* concentrations, and performed image processing to obtain the quantitative relationship as shown in **Figure 3a**. The detection limit of *E. coli* was obtained to be 3.38×10^2^ CFU/mL. It is worth mentioning that the detection system showed a good linear relationship within 10^3^-10^9^ (Y = 0.15X-0.36; R^2^ = 0.98). A fluorescent enzyme-linked immunosorbent assay based on SS-DNAt using a streptavidin scaffold DNA tetramer (SS-DNAt) was designed by Jing et al. The method improved the sensitivity (10^4^ ~ 10^6^ CFU/mL) for the detection of *E. coli* O157:H7 in milk samples (Jing et al., 2023). Meanwhile, Fang et al. achieved the detection of *E. coli* by enhancing the fluorescence through synthesized Ag^+^-doped gold nanoclusters (AgAuNC) and then quenching the fluorescence using a combination of I^2^ and I^-^, with a linear range of 3.3 × 10^3^ ~ 10^6^ CFU/mL, and a limit of detection of 9.2 × 10^2^ CFU/mL (Fang et al., 2022). Obviously, our method is simple and economical, and at the same time has a good sensitivity (**Table 2**).

**Table 2:** Comparison of AG-AI detection system detection system with other detection methods.

Method	Detection range (CFU/mL)	LOD (CFU/mL)	Reference
Based on SS-DNAt, fluorescence ELISA	10^4^ to 10^6^	3.75 × 10^3^	(Jing et al., 2023)
I_2_/I^−^quench fluorescence-enhanced Ag-AuNC in ELISA	3.3 × 10^3^ to 10^6^	9.2 × 10^2^	(Fang et al., 2022)
Based on field effect transistor	NO date	NO date	(Li et al., 2022)
immunofluorescence biosensor	10^3^ to 10^8^	10^3^	(Liu et al., 2021)
fluorescence biosensor	10^3^ to 10^9^	3.38 × 10^2^	This work

In the study of the specificity of this system, we chose *Acinetobacter baumannii* (bm), *Pseudomonas aeruginosa* (pae) and *Klebsiella pneumoniae* (kpn) as controls (10^7^ for *E. coli* and 10^9^ for the other bacteria). The results, as shown in **Figure 3b**, showed that the signal values of *E. coli* were significantly higher than those of the other bacteria, demonstrating the excellent specificity of our system.

### 3.3 Comparison of AG-AI detection system and matrix-assisted laser desorption ionization/time-of-flight mass spectrometry

**Figure 4:**
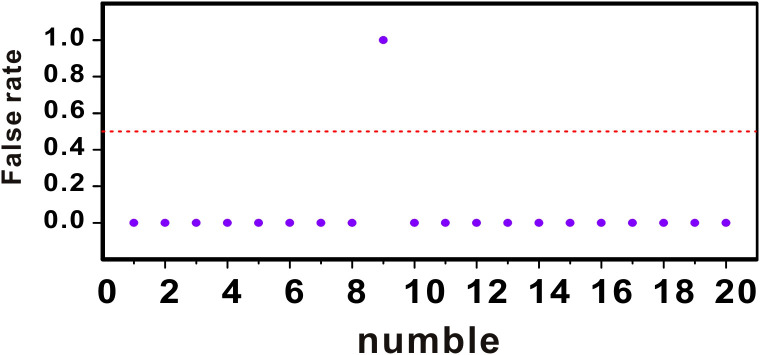
Comparison of MALDI-TOF MS and AG-AI detection system.

In order to verify the accuracy of the AG-AI detection system, we used the AG-AI detection system and MALDI-TOF MS to test these 20 specimens simultaneously. The samples were diluted into different concentrations of *E. coli* bacterial fluids using saline. The results are shown in **Figure 4**. The AG-AI system did not match the MALDI-TOF MS for only one specimen, which proved that the AG-AI system has good accuracy.

## 4 Discussion

Urinary tract infections are one of the common routes of bacterial infection in humans. The dry chemical test strips are usually used in the initial diagnosis of urinary tract infections. However, the sensitivity of test strips is low, and false-negative results may appear on urine test strips in the presence of non-nitrite-producing pathogens. The recognized gold standard for the diagnosis of bacterial urinary tract infections is *in vitro* urine culture (Capstick et al., 2024), but the identification of cultured bacteria is completely dependent on the experience of the examiner. Misdiagnosis often occurs due to lack of experienced testing personnel, and judgment may vary between personnel. Mass spectrometry is a rapidly developing method of identifying bacteria with high accuracy in recent years. However, mass spectrometry equipment is too expensive to afford it for many small hospitals. Thus, there is a urgent need for a method of high-sensitivity and low-cost in the diagnosis of urinary tract infection.

In this study, the AG-AI detection system was constructed. The AG-AI detection system has good sensitivity (10^3^-10^9^ CFU/mL) and high specificity, in which *E. coli* can be effectively distinguished from other bacteria. Meanwhile, we further validated the consistency of this detection system with time-flight mass spectrometry. Surprisingly, the results of the assay system were in high agreement with the time-flight mass spectrometry, which proved that the AG-AI assay system has good accuracy. This study demonstrates that the AG-AI detection system can effectively assist the testing staff in identifying uropathogenic bacteria. In addition, compared with mass spectrometry, the AG-AI system is more economical and rapid. These characteristics make the AG-AI system widely used in most economically disadvantaged areas and hospitals.

In recent years, various methods of supplementary diagnosis have been reported. Liu et al. used antibody-modified Zn_2_GeO_4_: Mn nanoparticles (ZGO: Mn NPs) to capture *E. coli* and *Staphylococcus aureus* in urine by identifying luminescent dots from the raw images and converting them to digital signals by a machine vision algorithm. The sensitivity of the method was 10^3^ ~ 10^8^ CFU/mL and the detection limit was 10^3^ CFU/mL (Liu et al., 2021). Kim et al. developed a urine multi-marker sensor capable of measuring biomarkers from urine (Kim et al., 2021). The relationship between clinical outcomes and a set of sensing signals for four different prostate cancer (PCa) biomarkers were analyzed by two different machine learning (ML) algorithms. Wityk et al. combined optical methods with machine learning, testing 27 algorithms and achieving an accuracy of 97%. Their approach is comprehensive and reliable (Wityk et al., 2023). Ma et al. identified *E. coli* microcolony stage targets by incubating the microorganisms for 3 h. The results of this study are presented in the following paper (Ma et al., 2022). Combined with phase contrast microimaging, *E. coli* was distinguished from seven other common foodborne bacteria by artificial intelligence with an average accuracy of 94%. The method was also applicable at *E. coli* concentrations greater than three orders of magnitude with an R^2^ of 0.995. All of research studies confirmed the feasibility and value of AI applied in clinical diagnosis.

In contrast to other similar studies, our detection system could specifically capture *E. coli* by the specific aptamers modified with GQDs, followed by image processing by AI. The principle of detection is that the fluorescence emitted by the GQDs on the slide showed a positive correlation with the concentration of *E. coli*. Therefore, the AG-AI detection system can realize the sensitive detection and accurate identification of *E. coli*. We believe that AG-AI detection system has several advantages as follows; 1. The detection system is cost effective (GQDs and aptamers used in this system are cheap). Some small hospitals cannot be equipped with mass spectrometers due to limited funds; AG-AI detection system provides effective support for inspectors in detecting urinary tract infections and bacterial identification. 2. Bacterial identification requires experienced inspectors, which requires high cultivation requirements for inspectors. And there are differences in the judgement of the results between different inspectors. AG-AI detection system effectively eliminates the influence of personnel differences on the results through objective data processing and analysis. 3. AG-AI detection system is an effective method for identifying bacteria, which makes up for the shortcomings of the current methods for identifying bacteria. The method can assist inspectors to identify bacteria and is also an effective supplement to mass spectrometry analyzers. 4. Theoretically, the AG-AI detection system can achieve identification of different bacteria by replacing different aptamers. Different aptamers can be selected according to the needs of identification of bacteria.

This study also has some limitations. 1) The small sample size of the experiment will affect the final performance of the AG-AI detection system. 2) This study addresses the methodology construction for the detection of *E. coli*, But the identification of other bacteria has not yet been addressed. The above points will be further improved in future studies.

## 5 Conclusion

In this study, we constructed the AG-AI detection system, which can detect *E. coli* with high sensitivity and good specificity. This AG-AI detection system has several unique advantages: (i) The detection system has a wide linearity (10^3^-10^9^ CFU/mL) and a low detection limit (3.38×10^2^ CFU/mL) for the detection of *E. coli*. (ii) The AG-AI detection system can effectively differentiate between *E. coli* and other urinary tract infection bacteria (bm, pae, and kpn). (iii) The AG-AI detection system is in good agreement with the method of MALDI-TOF MS. (iv) This method is a reliable assistant for clinical microbiological examiners. (v) Surprisingly, we can detect different causative bacteria of urinary tract infection by changing different aptamers.

## Data Availability

The original contributions presented in the study are included in the article/**Supplementary Material**. Further inquiries can be directed to the corresponding authors.
