# Supplementary material for: Aptamer-functionalized graphene quantum dots combined with artificial intelligence detect bacteria for urinary tract infections
Source: Front Cell Infect Microbiol. 2025 Apr 16;15:1555617. doi: 10.3389/fcimb.2025.1555617 (PMC12040687; doi:10.3389/fcimb.2025.1555617)
Supplement: Supplementary file 1 [file DataSheet1.docx]

**Supporting Information**

Aptamer-functionalized graphene quantum dots combined with Artificial intelligence detect bacteria for urinary tract infections

Kun Li ^1^, Shiqiang Fang ^1^, Tangwei Wu ^1^, Chao Zheng ^1^, Yi Zeng ^1^, Jinrong He ^2^, Yingmiao Zhang ^1, 3^*, Zhongxin Lu ^1, 2,^ *

^1^ Department of Medical Laboratory, The Central Hospital of Wuhan, Tongji Medical College, Huazhong University of Science and Technology Wuhan, China

^2^ Cancer Research Institute of Wuhan, The Central Hospital of Wuhan, Tongji Medical College, Huazhong University of Science and Technology Wuhan, China

^3^ Hubei Provincial Engineering Research Center of Intestinal Microecological Diagnostics, Therapeutics, and Clinical Translation, Wuhan, China

* Correspondence:

Yingmiao Zhang, zhangyingmiao@zxhospital.com

Zhongxin Lu, luzhongxin@zxhospital.com.

**Construction of Artificial Intelligence Model:** An OpenCV algorithm was applied to be trained and detect the fluorescent image. The dataset used is a self-constructed dataset with the number of 100 samples. The dataset needs to be pre-processed, the resolution of the dataset images is converted to the same size, and the data is collected under the same light conditions to avoid the influence of light brightness. The image data need to be grayed out, filtered and other preprocessing. In constructing the model, the dataset of this article is 100 samples, of which, 70 samples are sample training collection, and 30 samples are validation collection. The result of validation collection of 30 samples is shown in Fig S1, which yields an accuracy of 96.7%.





Fig S1 The accuracy of AG-AI detection system.


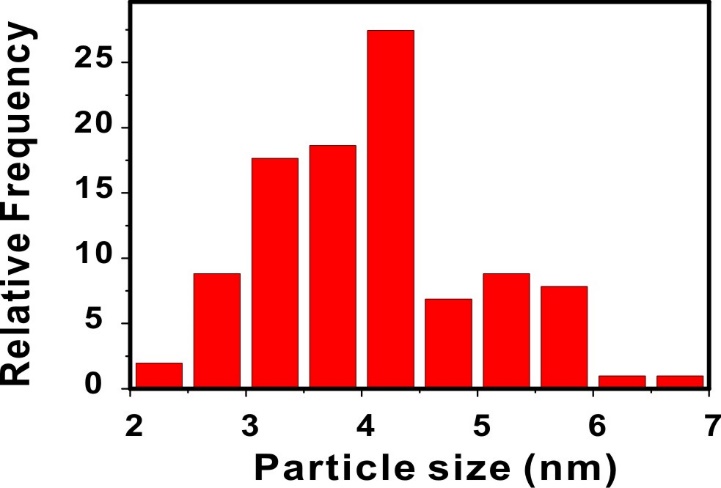


Fig S2. The particle size distribution of GQDs.
